# Chromosome Segregation Analysis in Human Embryos Obtained from Couples Involving Male Carriers of Reciprocal or Robertsonian Translocation

**DOI:** 10.1371/journal.pone.0046046

**Published:** 2012-09-27

**Authors:** Ahmet Yilmaz, Xiao Yun Zhang, Jin-Tae Chung, Seang Lin Tan, Hananel Holzer, Asangla Ao

**Affiliations:** 1 Department of Obstetrics and Gynecology, McGill University, Montreal, Quebec, Canada; 2 Department of Human Genetics, McGill University, Montreal, Quebec, Canada; The Chinese University of Hong Kong, Hong Kong

## Abstract

The objective of this study was to investigate the frequency and type of chromosome segregation patterns in cleavage stage embryos obtained from male carriers of Robertsonian (ROB) and reciprocal (REC) translocations undergoing preimplantation genetic diagnosis (PGD) at our reproductive center. We used FISH to analyze chromosome segregation in 308 day 3 cleavage stage embryos obtained from 26 patients. The percentage of embryos consistent with normal or balanced segregation (55.1% vs. 27.1%) and clinical pregnancy (62.5% vs. 19.2%) rates were higher in ROB than the REC translocation carriers. Involvement of non-acrocentric chromosome(s) or terminal breakpoint(s) in reciprocal translocations was associated with an increase in the percent of embryos consistent with adjacent 1 but with a decrease in 3∶1 segregation. Similar results were obtained in the analysis of nontransferred embryos donated for research. 3∶1 segregation was the most frequent segregation type in both day 3 (31%) and spare (35%) embryos obtained from carriers of t(11;22)(q23;q11), the only non-random REC with the same breakpoint reported in a large number of unrelated families mainly identified by the birth of a child with derivative chromosome 22. These results suggest that chromosome segregation patterns in day 3 and nontransferred embryos obtained from male translocation carriers vary with the type of translocation and involvement of acrocentric chromosome(s) or terminal breakpoint(s). These results should be helpful in estimating reproductive success in translocation carriers undergoing PGD.

## Introduction

Approximately 9% of all couples world-wide experience infertility [1]. Male infertility plays a role in half of these infertility cases and may result from congenital or environmental factors such as infections, and testicular, endocrine, or genetic abnormalities [2], [3]. Chromosome translocations are an important genetic cause of male infertility [4], [5].

Balanced translocations are among the most common chromosomal abnormalities seen in humans [6]–[9]. Approximately 0.2% of all live births carry a translocation and their frequency is further increased among infertile men [5], [10], [11]. Translocations typically occur as a result of exchange of chromosomal material between two non-homologous chromosomes (reciprocal translocations, REC) or centric fusion of long arms of two non-homologous acrocentric chromosomes (Robertsonian translocation, ROB). Most translocation carriers are phenotypically normal because there is no net loss or gain of genetic information [12], [13]. However, chromosome segregation during gametogenesis in these individuals may lead to production of gametes with a lack or excess of genetic material [14]. The majority of gametes, and consequently embryos, produced by these individuals are genetically abnormal, resulting in infertility, miscarriages, or in some cases birth of a child with genetic abnormalities [5], [15], [16].

Analysis of chromosome segregation patterns in embryos obtained from couples involving male translocation carriers may help in understanding mechanisms important in chromosome segregation and predicting future chances of healthy pregnancy in these couples. Preimplantation genetic diagnosis (PGD), genetic analysis of embryos before implantation to select and transfer only genetically normal embryos, provides a unique opportunity to analyze chromosome segregation patterns in translocation carriers before implantation. The objective of this study was to investigate the frequency and type of chromosome segregation patterns and their clinical outcome in cleavage stage embryos obtained from ROB and REC male translocation carriers undergoing PGD at our reproductive center.

## Materials and Methods

### Patients

Male patients with REC (n = 15) or ROB (n = 11) underwent 42 PGD cycles (26 REC and 16 ROB cycles) at the McGill University Health Center (MUHC)-Reproductive Center located in Montreal, Quebec, Canada (Tables 1 and 2). The female partners did not have any known genetic abnormality. All couples had signed consent forms for PGD at the MUHC Reproductive Center. This project was approved by the Royal Victoria Hospital-MUHC Ethics Board.

**Table 1:** Karyotype and reproductive history of the male translocation carriers.

Patient	Translocation type	PGD cycles	Female age^a^	Male age	Sperm parameters^b^	Reproductive history
1	46,XY,t(1;3)(q42.1;p25)	1	37	37	Normal	2 TOP
2	46,XY,t(1;7)(p36.1;q11.23)	2	38	42	OS	Primary infertility
3	46,XY,t(1;15)(p36.2;q14)	2	35	35	Normal	3 miscarriages
4	46,XY,t(1;16)(p31.3;q23.2)^c^	2	29.5	34	Normal	Primary infertility
5	46,XY,t(2;4)(q33.1;q35)	1	35	38	TRT	1 miscarriage
6	46,XY,t(3;4)(q26.2;p15.3)	4	35.3	36	AST	Primary infertility
7	46,XY,t(3;4)(q27;p14)	2	36.5	40	OS	Primary infertility
8	46,XY,t(3;7)(q26.1;q35)	1	30	36	Normal	3 TOP (affected)
9	46,XY,t(5;9)(p13;p24)	2	33	39	Normal	2 miscarriages
10	46,XY,t(9;12)(q12;p12.2)	1	32	37	AST	Primary infertility
11	46,XY,t(9;12)(p23;q14)	1	42	40	OS	Primary infertility
12	46,XY,t(9;15)(p24;q11.2)	2	35	40	OS,AST,TRT	Primary infertility
13	46,XY,t(11;22)(q23.3;q11.2)	2	36.5	36	AST	Secondary infertility
14	46,XY,t(11;22)(q23.3;q11.2)	1	33	33	Normal	Primary infertility
15	46,XY,t(12;15)(p10;p10)	2	32.5	33	OS	2 TOP
16	45,XY,der(13;14)(q10;q10)	1	29	31	OS	Primary infertility
17	45,XY,der(13;14)(q10;q10)	1	33	33	OS, AST	Primary infertility
18	45,XY,der(13;14)(q10;q10)	1	27	31	OS	Primary infertility
19	45,XY,der(13;14)(q10;q10)	1	38	36	OS	Primary infertility
20	45,XY,der(13;14)(q10;q10)	3	32.3	33	OS	Primary infertility
21	45,XY,der(13;14)(q10;q10)	1	36	38	OS	1 TOP
22	45,XY,der(13;14)(q10;q10)	1	29	36	OS	Primary infertility
23	45,XY,der(13;15)(q10;q10)	1	32	33	Normal	1 miscarriage
24	45,XY,der(13;21)(q10;q10)	3	30	40	OS	Primary infertility
25	45,XY,der(13,21)(q10;q10)	2	37.2	42	AST	1 miscarriage
26	45,XY,der(13;21)(q10;q10)	1	32	35	OS	Primary infertility

a Age of patient at the time of embryo biopsy averaged over the number of PGD cycles the couple undergone.

b OS = oligospermia, AST = asthenospermia, TRT = teratozoospermia, TOP = termination of pregnancy.

c This couple used donor eggs from 32 and 27 year old women in the two PGD cycles.

**Table 2:** Clinical details of patients participated in this study categorized by the type of translocation^a^.

Clinical details	REC	ROB
Number of patients	15	11
Average age of the male carrier ± SD	37.1±2.7	35.3±3.6
Average age of the female partner ± SD	34.7±3.2	32.3±3.5
Number of cycles	26	16
Retrieved oocytes (per cycle)	438 (16.8)	262 (16.4)
Zygotes with two pronuclei (per cycle)	267 (10.3)	130 (8.1)
Embryos frozen	48	0
Embryos survived thawing	45	0
Embryos biopsied	235	115
Embryos with nuclei (% of embryos biopsied)	220 (94%)	105 (91%)
Embryos with valid FISH results (per cycle)	210 (8.1)	98 (6.1)
Embryos diagnosed as chromosomally normal or balanced (% of embryos with valid FISH results)	57 (27.1%)*	54 (55.1%)
Embryos transferred (% of normal embryos)	34 (59.6%)	29 (53.7%)
Cycles with at least one sac	5	10
Cycles with at least one fetal heart beat	4	9
Clinical pregnancy rate	19.2%*	62.5%
Implantation rate	14.7%*	41.4%

a Abbreviations used: REC = reciprocal translocation, ROB = Robertsonian translocation, an asterisk (*) indicates statistical difference at *P*<0.05 level.

### Reproductive history of male translocation carriers

Nineteen of the 26 patients suffered from various forms of sperm abnormalities including oligospermia, asthenospermia, and teratozoospermia (Table 1). Seven of the patients had normal sperm parameters but their partners suffered from infertility, miscarriages, or termination of pregnancies before opting for IVF and PGD. Fertilization was achieved using eggs obtained from female partners of the translocation carriers except that the patient with the 46,XY,t(1;16)(p31.3;q23.2) karyotype opted for egg donations from 32 and 27 year old women in the first and second cycles, respectively. Clinical pregnancy rate was defined as the number of cycles where at least one sac was observed four weeks after embryo transfer divided by the total number of cycles. Implantation rate was defined as the total number of sacs divided by the total number of embryos transferred.

### FISH

Single blastomeres were biopsied on day 3 post-insemination and spread on glass slides coated with poly-L-lysine and processed as described previously [17]. Centromeric probes (CEP), in combination with subtelomeric Telvysion probes, encompassing the translocated region were used in REC cases (Supplemental Table 1). In ROB cases, probes detecting the q arms of the two fused chromosomes were used. Two or three rounds of hybridization were performed based on the karyotype and the probe combinations used. The probes were purchased from Intermedico, a Canadian distributor of Abbott Molecular products, and Rainbow Scientific (Windsor, CT).

The nuclei and probe mixture were co-denatured at 75°C for 5 min and then hybridized in a moist chamber overnight at 37°C for the first round. The unbound probes were removed by stringent washing at 70°C for 2 minutes in 0.7 or 0.4×SSC (standard saline citrate solution) following the manufacturer's instructions. The slides were rinsed in 2×SSC-0.1% Tween20 at room temperature and the preparations were mounted in antifade solution (p-phenylenediamide dihydrochloride; Vector, Burlingame, CA) or DAPI nuclear stain and observed under a fluorescence microscope (Olympus BX60, Olympus Canada, Ontario, Canada). For the second and third rounds of FISH, slides were washed in 2×SSC and dehydrated in a series of alcohol following the same procedure used in the first round except that antifade containing DAPI (0.25 ng/ml) (Sigma, Oakville, Canada) was used as the counterstain. The embryos diagnosed as normal or balanced were transferred on day 4 or 5 post-insemination. The chromosome analysis was also performed on spare embryos (i.e., embryos that were not suitable for transfer or freezing) whenever patients donated them for research using the same FISH probes. It was not possible to distinguish between normal and balanced embryos in this study because we did not use probes that spanned the breakpoints.

Based on FISH results, embryos were classified as normal or balanced (diploid number of chromosomes), unbalanced, mosaic (embryos with normal/balanced cells mixed with unbalanced forms), chaotic (random loss or gain of chromosomes), or polyploid (multiple sets of chromosomes). Details of criteria used for embryo classification can be found elsewhere [18]. Classification of embryos was based only on translocated chromosomes unless otherwise indicated. Embryos that did not fit in any known segregation pattern were classified under the “no segregation” group. One of the ROB and two of the REC embryos were classified as chaotic on day 3 because the embryos contained multiple nuclei with apparently random loss or gain of chromosomes. The method previously described by Ye et al. [19] was used to determine if a translocation involved any terminal breakpoints. The distance between the telomere and the breakpoint was measured using ideograms provided by Gardner and Sutherland [20]. Data were analyzed using F-test for continuous and chi-square or Fisher's exact test for categorical data. Statistical significance level was set at *P* = 0.05.

## Results

We analyzed data obtained from 15 and 11 patients with REC and ROB, respectively, in 42 PGD cycles (Table 1). Nineteen of the 26 patients (73.1%) had abnormal sperm parameters including oligospermia, asthenospermia, and teratozoospermia. The female partners suffered from primary infertility (n = 16), miscarriages (n = 5), termination of pregnancies (n = 4), or secondary infertility (n = 1).

In our study, chromosomes such as 1, 3, 4, 9, 12, and 15 were most frequently involved in REC cases. Chromosome combinations 3;4, 9;12, and 11;22 were each involved in two REC cases. Chromosome 13, which was not involved in any of the REC cases, was involved in all of the ROB cases. Chromosome 14 and 21 were involved in seven and three ROB cases, respectively. Chromosome combinations 13;14 and 13;21 were involved in seven and three cases, respectively.

**Figure 1:**
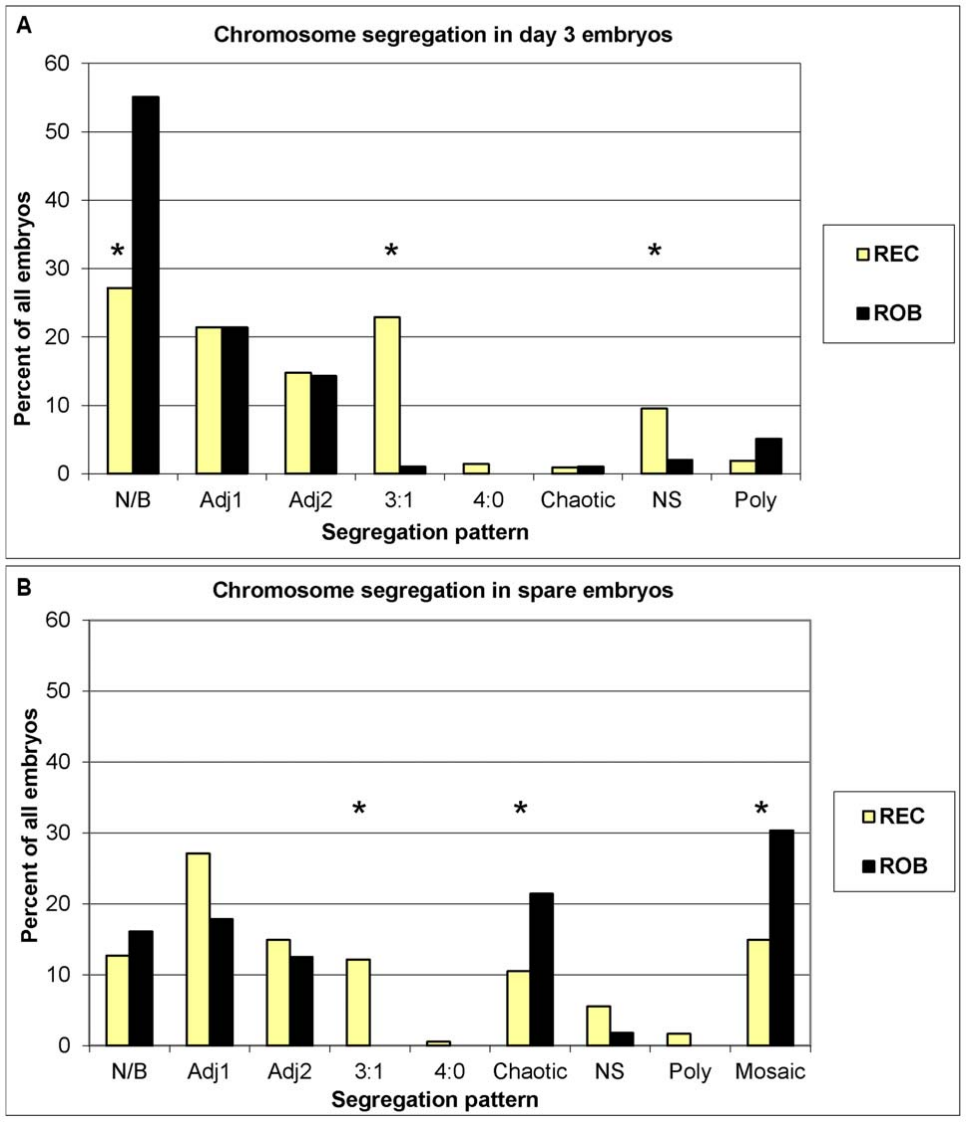
Chromosome segregation in day 3 cleavage stage and spare embryos obtained from male translocation carriers^a^. ^a^REC = reciprocal translocation; ROB = Robersonian translocation, N/B = chromosomally normal or balanced for the translocated chromosomes, Adj1 = adjacent 1, Adj2 = adjacent 2, NS = no known segregation pattern detected, Poly = polyploid. An asterisk (*) denotes statistically significant differences (*P*<0.05). The column labeled with “3∶1” represents frequency of 3∶0 segregants in ROB and 3∶1 in REC. Normal/balanced spare embryos in ROB and REC carriers were not compared.

The average age of the male patients and their female partners in each of the translocation categories were not statistically different (*P*>0.34, Table 2). On average, 16.8 and 16.4 oocytes from partners of REC and ROB carriers, respectively, were recovered in each cycle. Four of the 210 embryos obtained from the REC (1.9%) and three of the 98 embryos obtained from the ROB (3.1%) carriers did not give any valid FISH results on day 3. In addition, no nuclei were found in 14 REC (6.7%) and six (6.1%) ROB embryos. These embryos without any nuclei or valid FISH results were excluded from further analysis. The percent of embryos diagnosed as chromosomally normal or balanced (27.1% vs. 55.1%), and clinical pregnancy (19.2% vs. 62.5%) and implantation (14.7% vs. 41.4%) rates were higher in ROB than the REC carriers despite similar female ages in the two groups of patients (Table 2). Patients with reciprocal translocations involving at least one non-acrocentric chromosome or terminal breakpoint produced more embryos consistent with adjacent 1 but fewer embryos with 3∶1 segregation (Table 3). Percent segregation patterns in day 3 embryos with valid FISH results are presented in Figure 1A. In both types of translocation, alternate segregation was the most frequent mode of segregation (27.1% in REC and 55.1% in ROB) and adjacent 1 was more frequent than adjacent 2. Percent embryos without any known segregation pattern was higher in REC than ROB (9.5% vs. 2%). We also performed FISH on spare REC (n = 181) and ROB (n = 56) embryos that were not suitable for transfer or freezing and donated for research (Figure 1B). Adjacent 1 segregation was more frequent than adjacent 2 in spare embryos in both REC and ROB carriers. The proportion of spare embryos with mosaic (30.4% vs.14.9%) and chaotic (21.4% vs. 10.5%) segregation were approximately twice higher in ROB than the REC carriers. Comparisons of segregation patterns in day 3 and spare embryos are presented in Supplemental Figures S1A and S1B. In REC carriers, percent 3∶1 segregation was higher in day 3 than the spare embryos. The percentage of embryos that did not show any known segregation pattern tended to be higher in day 3 than the spare embryos (*P* = 0.08). In ROB carriers, percent embryos consistent with adjacent 1 or adjacent 2 in day 3 and spare embryos were similar.

**Table 3:** Percent segregation and number of embryos (in parenthesis) obtained from reciprocal translocation carriers classified based on presence of chromosomes with terminal breakpoints or acrocenteric chromosomes^a^.

	Day 3		Spare		Day 3		Spare	
	ACR	NonACR	ACR	NonACR	TER	NonTER	TER	NonTER
N/B	27.7 (18)	26.9 (39)	15.5 (9)	11.4 (14)	28.4 (31)	25.7 (26)	14.6 (13)	10.9 (10)
Adjacent 1	13.8 (9)	24.1 (35)	22.4 (13)	29.3 (36)	23.9 (26)	18.8 (19)	33.7 (30)	20.7 (19)
Adjacent 2	15.4 (10)	14.5 (21)	15.5 (9)	14.6 (18)	16.5 (18)	12.9 (13)	15.7 (14)	14.1 (13)
3∶1	32.3 (21)	19.3 (28)	20.7 (12)	8.1 (10)	17.4 (19)	28.7 (29)	10.1 (9)	14.1 (13)
4∶0	0 (0)	2.1 (3)	0 (0)	0.8 (1)	0.9 (1)	2.0 (2)	0 (0)	1.1 (1)
NS	7.7 (5)	10.3 (15)	5.2 (3)	5.7 (7)	9.2 (10)	9.9 (10)	7.9 (7)	3.3 (3)
Mosaic	N/A	N/A	10.3 (6)	17.1 (21)	N/A	N/A	5.6 (5)	23.9 (22)
Chaotic	3.1 (2)	0 (0)	8.6 (5)	11.4 (14)	1.8 (2)	0 (0)	10.1 (9)	10.9 (10)
Polyploid	0	2.8 (4)	1.7 (1)	1.6 (2)	1.8 (2)	2.0 (2)	2.2 (2)	1.1 (1)
**TOTAL** b	**65**	**145**	**58**	**123**	**109**	**101**	**89**	**92**

a Abbreviations used: ACR = acrocentric chromosome(s) involved; NonACR = no acrocentric chromosome(s) involved; TER = chromosome(s) with terminal breakpoint(s) involved; NonTER = no chromosome(s) with terminal breakpoint(s) involved; N/B = embryos consistent with normal or balanced chromosome complement; NS = embryos without any known segregation type; N/A = not applicable.

b Total number of embryos.

## Discussion

Chromosome translocations are an important cause of male infertility [5], [11], [21]. The determination and comparison of segregation patterns in embryos obtained from the male REC and ROB carriers are useful in determining the risk of infertility, chromosomally abnormal child or recurrent miscarriages [22]–[24], [25], [26]. However, a systematic review of the literature shows that such data comparing segregation in REC and ROB embryos obtained from the same center are very limited in the English language journals [22], [27]. The objective of this study was, therefore, to examine frequency and clinical relevance of chromosomal segregation patterns in day 3 and spare embryos obtained from male REC and ROB carriers who opted for PGD at our reproductive center.

We analyzed data obtained from 308 day 3 cleavage stage and 237 spare embryos collected from 15 REC and 11 ROB male carriers in 42 PGD cycles (Table 1). The presence of sperm abnormalities were common (73.1%) in this cohort of patients, confirming previous findings [28], [29]. The percent chromosomally normal or balanced embryos (27.1% vs. 55.1%), and pregnancy (19.2% vs. 62.5%) and implantation (14.7% vs. 41.4%) rates were higher in ROB than the REC carriers despite similar maternal age (*P* = 0.70) and percentage of normal/balanced embryos transferred (59.6% vs. 53.7%). Most studies in the literature report clinical pregnancy rates calculated based on detection of fetal heart beats. The pregnancy rates based on positive fetal heart beats in our study were 15.4% in REC and 56.3% in ROB carriers. Pregnancy rates in other studies based on fetal heart beats range from 13.1% to 27.3% in REC [22], [30] and 14.3% to 33% in ROB carriers [22], [30], [31]. One of the main reasons for this large variation in pregnancy rates in translocation carriers could be that the sample size in most of the segregation studies, including ours, may not be sufficient to draw any strong conclusions on pregnancy rates [22], [24], [32], [33]. Despite this limitation, we believe there were two main reasons for the higher clinical pregnancy rates obtained in ROB carriers in our study. First, ROB carriers produced more normal or balanced embryos than the REC carriers (27.1% vs. 55.1%), providing us with more options to select embryos that were not only chromosomally normal but also morphologically of better quality. Although publications comparing segregation in a reasonably large number of REC and ROB embryos from the same center are very rare, the comparison of data obtained from different centers show that the ROB carriers produce a higher percent normal or balanced embryos. The published percent of normal or balanced embryos range from 18% to 43% in REC [5], [23], [32] whereas it is 28% to 77% in ROB carriers [5], [22], [28]. The second reason for the higher pregnancy rates obtained in ROB carriers could be that we transferred embryos normal or balanced for not only translocated but also for some of the nontranslocated chromosomes. We screened ROB embryos using the polar body probe panel (Abbott Molecular, Downers Grove, IL) that included probes for chromosomes 13, 16, 18, 21, and 22, some of which were not translocated in some of our patients (Supplemental Table 1). Eleven of the day 3 ROB embryos (20.3% of all normal embryos) were normal or balanced for translocated but aneuploid for the nontranslocated chromosomes and were not used for transfer.

In an attempt to investigate possible effects of location of breakpoints and chromosome type on meiotic segregation patterns in the male REC carriers, we classified the segregation data obtained from the day 3 cleavage stage embryos based on the presence of acrocentric chromosomes or chromosomes with terminal breakpoints involved in the translocation. Involvement of non-acrocentric chromosome(s), or terminal breakpoint(s) in REC was associated with an increase in the percent of embryos consistent with adjacent 1 but with a decrease in 3∶1 segregation although the percentage of normal or balanced embryos was largely unaffected (Table 3). Similar results were obtained in the analysis of spare embryos. Re-analysis of the male data obtained from a study published recently [19] also showed no change in percent normal or balanced (15% vs. 13%), higher adjacent 1 (24.6% vs. 35%) and lower 3∶1 (40% vs. 27%) segregation in translocations with non-acrocentric chromosomes.

Chromosome segregation patterns in day 3 cleavage stage embryos are presented in Figure 1. Excluding 3∶1, the most frequent type of segregation was alternate followed by adjacent 1 and adjacent 2 in both REC and ROB carriers, results in agreement with previously published studies [22], [33]. The frequency of alternate and 3∶1 segregation in REC carriers, on the other hand, varies substantially among published studies. The percent alternate segregation ranges from 13.9% to 43.2% in the current literature [19], [22]–[24], [32]–[34]. Three to one segregation was the most frequent type of segregation in some of these studies [19], [22] whereas one of the two least frequent types in the others [23], [24], [34]. The frequency of alternate and 3∶1 segregants in REC carriers in our study does not agree with the findings of Mackie-Ogilvie et al. [32] and Ye et al. [19]. These discrepancies may be due to differences in sample size, embryos obtained per patient, patients' characteristics, location of translocation breakpoints, or the type of chromosomes involved.

The comparison of segregation patterns in day 3 and spare embryos is presented in Supplemental Table 2 and Supplemental Figures S1A and S1B. In REC carriers, 3∶1 segregation was more frequent on day 3 than in the spare embryos, suggesting that the 3∶1 segregation may have been erroneously overestimated on day 3. Diagnosis in day 3 and spare embryos were more similar in ROB than the REC carriers (Supplemental Figures S1A and S1B), which may be because of the fact that REC quadrivalents formed at pachytene may segregate in more diverse patterns [35].

Two of our REC patients were carriers of the t(11;22)(q23;q11), the only non-random REC with the same breakpoint reported in a large number of unrelated families [36], [37]. Analysis of 35 day 3 and 20 spare embryos obtained from these patients in three PGD cycles showed that 3∶1 segregation was the most frequent segregation type in both day 3 (31%) and spare (35%) embryos. Our results do not agree with Gardner and Sutherland [20] who reviewed segregation data in embryos obtained from four male carriers of this type of translocation and reported that only 2% of embryos were consistent with 3∶1 segregation. The reason for this discrepancy could be because of the small sample size or differences in the criteria used for classification of the embryos. Our results obtained from day 3 and spare embryos suggest that the birth of children with derivative chromosome 22 from these couples may be due to the high percent 3∶1 segregation found in these patients' embryos.

We detected higher rates of mosaicism in ROB than the REC spare embryos. Rates of chaotic or mosaic embryos are high, not only in translocation carriers, but also in regular IVF patients [27], [38]–[40]. Although the exact origin and nature of mosaicism are still subject to debate, theories that have been put forward to explain the causes of this phenomenon include nondisjunction combined with anaphase lag [41], mitotic errors due to lack of cell cycle checkpoint controls [17], [38], [42], or unknown patient-specific factors [41], [43].

In our cohort of patients, there was only one case of serious misdiagnosis, as defined by the embryo being diagnosed as normal or balanced on day 3 but unbalanced on day 5 when the spare embryo was analyzed. That misdiagnosed embryo was consistent with normal or balanced segregation on day 3 but was found to be consistent with adjacent 2 on day 5. The misdiagnosis may have occurred due to technical limitations of FISH such as signal overlaps, split signals, or loss of micronuclei [29]. In addition, it is possible that the embryo was consistent with alternate segregation on day 3 but converted into adjacent 2 on day 5 due to random mitotic errors.

Although we used FISH in this study, new DNA-chip based methodologies such as SNP microarrays and array-based comparative genomic hybridization (aCGH) have been rapidly gaining popularity for PGD of translocations [44], [45]. Both SNP microarrays and aCGH involve hybridization of fragmented and labeled DNA to short probes imprinted on solid surfaces. Both methods have been validated for clinical use and provide comprehensive information on the translocation status of the whole chromosomes in contrast to FISH which can examine only chromosome fragment(s) [44], [45]. In addition, analysis by the DNA-chip based methodologies may reduce the number of samples without any valid test results when analyzed by FISH. In our study, 3.1% and 1.9% of the embryos obtained from ROB and REC carriers, respectively, were excluded from the analysis because of poor hybridization. The SNP microarrays additionally offer DNA sequence data which may be used in more accurate evaluation of the breakpoints, uniparental disomy, or microdeletions [46].

In summary, our results show novel differences in chromosome segregation patterns in day 3 embryos obtained from male translocation carriers with different types of chromosomes and breakpoints involved. We confirmed some of these differences in spare embryos. We report a large difference in percent embryos consistent with normal or balanced segregation in ROB and REC carriers who underwent PGD at our reproductive center. We are not aware of any other segregation studies, with the sample size comparable to ours, conducted to investigate chromosome segregation in REC and ROB day 3 and spare embryos using samples from the same center. These results may prove valuable in the future because the newer techniques such as microarrays may be too costly and laborious to individually analyze a large number of cells in spare embryos. Future studies with larger sample size will hopefully lead to a better understanding of complex chromosome segregation patterns in embryos obtained from these patients and create alternative treatment options.

## Supporting Information

Figure S1 **Comparison of chromosome segregation in day 3 cleavage stage and spare embryos obtained from male translocation carriers^a^.** ^a^REC = reciprocal translocation; ROB = Robertsonian translocation, N/B = chromosomally normal or balanced for the translocated chromosomes, Adj1 = adjacent 1, Adj2 = adjacent 2, NS = no known segregation pattern detected, Poly = polyploid. An asterisk (*) denotes statistically significant differences (*P*<0.05). The column labeled with “3∶1” represents frequency of 3∶0 segregants in ROB and 3∶1 in REC. Normal/balanced and mosaic day 3 and spare embryos were not compared.
(TIF)

Table S1 **Probes used in FISH analysis^a^.**
(DOC)

Table S2 **Percentages and number (in parenthesis) of day 3 and spare embryos analyzed in this study^a^.**
(DOCX)
