# Supplementary figures and images for: Chromosome Segregation Analysis in Human Embryos Obtained from Couples Involving Male Carriers of Reciprocal or Robertsonian Translocation
Source: PLoS One. 2012 Sep 27;7(9):e46046. doi: 10.1371/journal.pone.0046046 (PMC3459837; doi:10.1371/journal.pone.0046046)

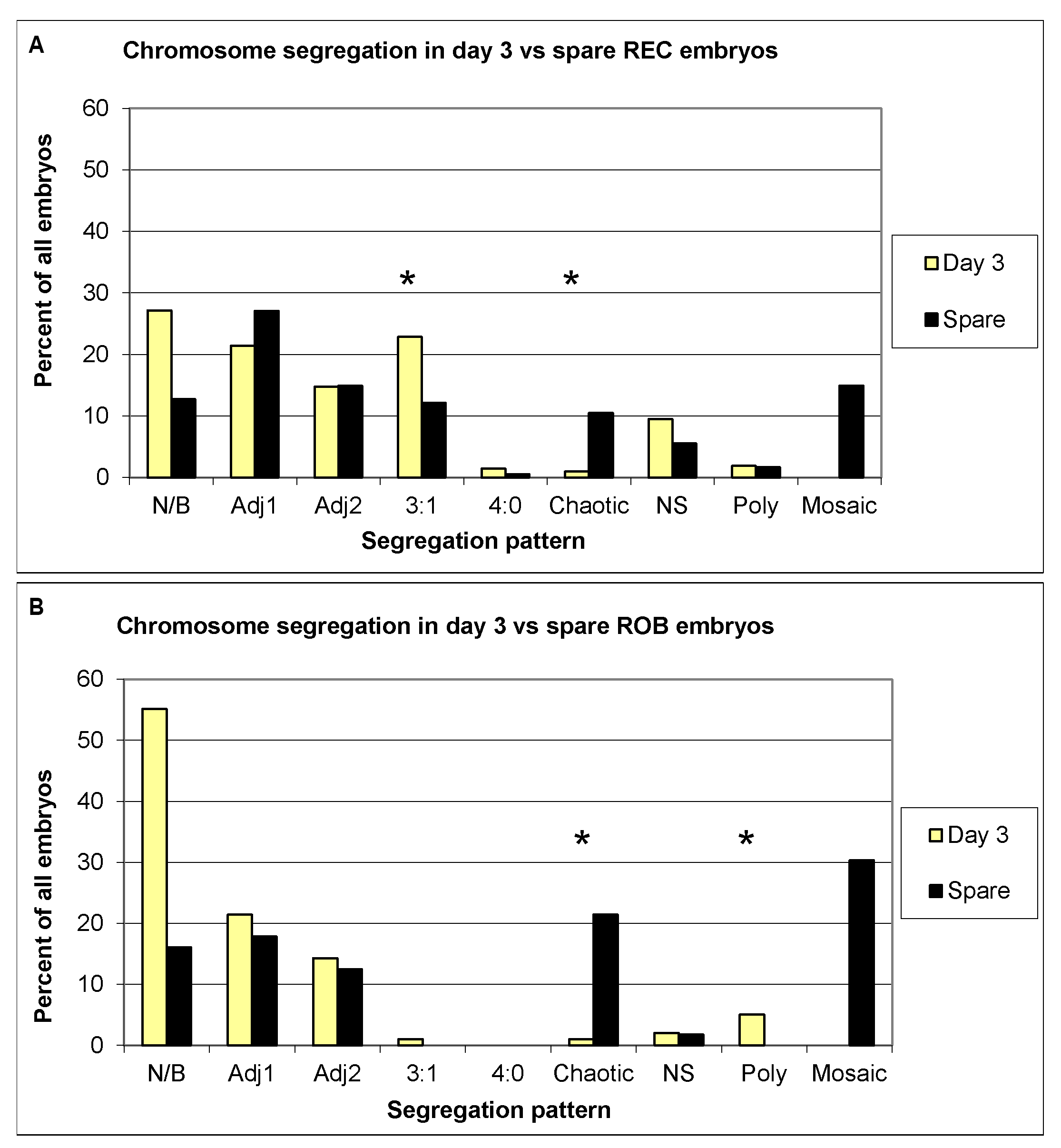

Supplement: Figure S1 — Comparison of chromosome segregation in day 3 cleavage stage and spare embryos obtained from male translocation carriersa. aREC = reciprocal translocation; ROB = Robertsonian translocation, N/B = chromosomally normal or balanced for the translocated chromosomes, Adj1 = adjacent 1, Adj2 = adjacent 2, NS = no known segregation pattern detected, Poly = polyploid. An asterisk (*) denotes statistically significant differences (P<0.05). The column labeled with “3∶1” represents frequency of 3∶0 segregants in ROB and 3∶1 in REC. Normal/balanced and mosaic day 3 and spare embryos were not compared. (TIF) [file pone.0046046.s001.tif]
