# Supplementary material for: Chromosome Segregation Analysis in Human Embryos Obtained from Couples Involving Male Carriers of Reciprocal or Robertsonian Translocation
Source: PLoS One. 2012 Sep 27;7(9):e46046. doi: 10.1371/journal.pone.0046046 (PMC3459837; doi:10.1371/journal.pone.0046046)
Supplement: Table S1 — Probes used in FISH analysisa. (DOC) [file pone.0046046.s002.doc]

| **Patient** | **Karyotype** | **First round** | **Second round** |
| --- | --- | --- | --- |
| **1** | 46,XY,t(1;3)(q42.1;p25)^b^ | Tel3p (g), Tel1q (o) | CEP3 (o) |
| **2** | 46,XY,t(1;7)(p36.1;q11.23) | Tel1p (g), Tel7q (o) | CEP7 (g), CEP1 (o) |
| **3** | 46,XY,t(1;15)(p36.2;q14) | Tel1p (g), Tel15q (o) | CEP15 (g),CEP1 (o) |
| **4** | 46,XY,t(1;16)(p31.3;q23.2) | Tel1p (g),Tel16q (o) | CEP1 (o),CEP16 (g) |
| **5** | 46,XY,t(2;4)(q33.1;q35)^c^ | Tel2q (o) | Tel4q (o) |
| **6** | 46,XY,t(3;4)(q26.2;p15.3) | Tel3q (o), Tel4p (g) | CEP3 (o), CEP4 (g) |
| **7** | 46,XY,t(3;4)(q27;p14) | Tel3q (o), Tel4p (g) | CEP3 (o), CEP4 (g) |
| **8** | 46,XY,t(3;7)(q26.1;q30)^d^ | Tel3q (o) | Tel7q (o) |
| **9** | 46,XY,t(5;9)(p13;p24) | Tel9p (g) | Tel5p (g), CEP9 (o) |
| **10** | 46,XY,t(9;12)(q12;p12.2) | Tel9q (o), Tel12p (g) | CEP9 (o),CEP12 (g) |
| **11** | 46,XY,t(9;12)(p23;q14) | Tel9p (g), Tel12q (o) | CEP9 (o),CEP12 (g) |
| **12** | 46,XY,t(9;15)(p24;q11.2) | Tel15q (o) | CEP9 (o), CEP15 (g) |
| **13** | 46,XY,t(11;22)(q23.3;q11.2) | Tel11q (o) | Tel22q (o), CEP11 (g) |
| **14** | 46,XY,t(11;22)(q23.3;q11.2) | CEP11 (g), Tel22q (o) | Tel11q (o) |
| **15** | 46,XY,t(12;15)(p10;p10)^e^ | LSI12 (g), LSI21 (o) | CEP15 (g) |
| **16** | 45,XY,der(13;14)(q10;q10) | PB | LSI14q32 (g), 18q21 (o) |
| **17** | 45,XY,der(13;14)(q10;q10) | PB | LSI14q32 (g), 18q21 (o) |
| **18** | 45,XY,der(13;14)(q10;q10) | PB | LSI14q32 (g), 18q21 (o) |
| **19** | 45,XY,der(13;14)(q10;q10) | PB | LSI14q32 (g), 18q21 (o) |
| **20** | 45,XY,der(13;14)(q10;q10) | LSI13(g) | LSI14 (g), CEP18 (o) |
| **21** | 45,XY,der(13;14)(q10;q10) | PB | LSI14q32 (g), 18q21 (o) |
| **22** | 45,XY,der(13;14)(q10;q10) | PB | LSI14q32 (g), 18q21 (o) |
| **23** | 45,XY,der(13;15)(q10;q10) | PB | CEP15 (g) |
| **24** | 45,XY,der(13;21)(q10;q10) | PB |  |
| **25** | 45,XY,der(13,21)(q10;q10) | PB |  |
| **26** | 45,XY,der(13;21)(q10;q10) | PB |  |

^a^Abbreviations used: g = green; o = orange; Tel = TelVysion; PB = polar body probe panel, a mixture of probes LSI13q14 (red), CEP16 (aqua), CEP18 (blue), LSI21 (green), and LSI22 (orange); CEP = chromosome enumerator probe; LSI = locus specific identifier.

^b^CEP1(o) was used in a third round of FISH

^c^CEP2 (o) and CEP4 (g) probes were used in a third round of FISH.

^d^CEP3 (o) and CEP7 (g) probes were used in a third round of FISH.

^e^Two different chromosome 15 probes D15Z1 (green) that covers 15p11.2 and D15Z4 (orange) that covers 15p11.1-q11.1 were used in the second and third rounds of FISH, respectively.
