# Supplementary material for: Chromosome Segregation Analysis in Human Embryos Obtained from Couples Involving Male Carriers of Reciprocal or Robertsonian Translocation
Source: PLoS One. 2012 Sep 27;7(9):e46046. doi: 10.1371/journal.pone.0046046 (PMC3459837; doi:10.1371/journal.pone.0046046)
Supplement: Table S2 — Percentages and number (in parenthesis) of day 3 and spare embryos analyzed in this studya. (DOCX) [file pone.0046046.s003.docx]

|  | Day 3 embryos | |  | Spare embryos | |
| --- | --- | --- | --- | --- | --- |
| Segregation pattern | REC | ROB |  | REC | ROB |
| Normal or balanced | 27.1 (57) | 55.1 (54) |  | 12.7 (23) | 16.1 (9) |
| Adjacent 1 | 21.4 (45) | 21.4 (21) |  | 27.1 (49) | 17.9 (10) |
| Adjacent 2 | 14.8 (31) | 14.3 (14) |  | 14.9 (27) | 12.5 (7) |
| 3:1 (REC) or 3:0 (ROB) | 22.9 (48) | 1 (1) |  | 12.2 (22) | 0 (0) |
| 4:0 | 1.4 (3) | N/A |  | 0.6 (1) | N/A |
| Mosaic | N/A | N/A |  | 14.9 (27) | 30.4 (17) |
| Chaotic | 1 (2) | 1 (1) |  | 10.5 (19) | 21.4 (12) |
| No segregation pattern | 9.5 (20) | 2 (2) |  | 5.5 (10) | 1.8 (1) |
| Polyploidy | 1.9 (4) | 5.1 (5) |  | 1.7 (3) | 0 (0) |
| TOTAL^b^ | 210 | 98 |  | 181 | 56 |

^a^REC = Reciprocal translocation; ROB = Robertsonian translocation;

N/A = not applicable.

^b^Total number of embryos.
